# Hydrogeological Conditions of a Crystalline Aquifer: Simulation of Optimal Abstraction Rates under Scenarios of Reduced Recharge

**DOI:** 10.1155/2013/606375

**Published:** 2013-12-17

**Authors:** Sandow Mark Yidana, Obed Fiifi Fynn, Larry Pax Chegbeleh, Prosper M. Nude, Daniel K. Asiedu

**Affiliations:** Department of Earth Science, University of Ghana, Legon, Accra, Ghana

## Abstract

A steady state numerical groundwater flow model has been calibrated to characterize the spatial distribution of a key hydraulic parameter in a crystalline aquifer in southwestern Ghana. This was to provide an initial basis for characterizing the hydrogeology of the terrain with a view to assisting in the large scale development of groundwater resources for various uses. The results suggest that the structural entities that control groundwater occurrence in the area are quite heterogeneous in their nature and orientation, ascribing hydraulic conductivity values in the range of 4.5 m/d to over 70 m/d to the simulated aquifer. Aquifer heterogeneities, coupled possibly with topographical trends, have led to the development of five prominent groundwater flowpaths in the area. Estimated groundwater recharge at calibration ranges between 0.25% and 9.13% of the total annual rainfall and appears to hold significant promise for large-scale groundwater development to support irrigation schemes. However, the model suggests that with reduced recharge by up to 30% of the current rates, the system can only sustain increased groundwater abstraction by up to 150% of the current abstraction rates. Prudent management of the resource will require a much more detailed hydrogeological study that identifies all the aquifers in the basin for the assessment of sustainable basin yield.

## 1. Introduction

Groundwater resources constitute arguably the most reliable buffer against the unremitting effects of climate change/variability and the concomitant ramifications on sustainable agriculture especially in the developing world. This is so because groundwater is mostly protected from high surface temperatures and the corresponding high evapotranspiration rates that affect surface flows and impoundments and thus render them ineffective sources of irrigation water supply to sustain large-scale irrigation activities. In the light of this, regional hydrogeological studies that lead to the development of aquifers for sustainable abstraction and management of groundwater resources is crucial.

There are various approaches available for regional hydrogeological investigations and for providing the necessary information required for optimal aquifer and basin yield management [1]. They include the application of remote sensing aerial photography, surface geological field investigations, application of advanced geophysical methods, and subsequent drilling to access aquifers. The conventional practice is the use of all data obtained from remote sensing techniques together with field based data and borehole information to develop a conceptual model which is then converted into a numerical model [2–11] to predict the hydrogeological conditions of aquifers. Basin wide hydrogeological investigations usually culminate in the development of regionally based numerical groundwater flow models which capture the essential aspects of the regional hydrogeology and thus provide decision support systems for the management of groundwater resources. The use of models will continue to provide useful leads to the effective management of flow and solute transport in aquifers, especially where climate change/variability and its attendant effects on the spatial and temporal variations in rainfall patterns place a huge uncertainty on the sustainability of rain-fed agricultural activities and where increasing global temperatures and low humidities render surface impoundments unsustainable as irrigation water sources. They are unavoidable in large-scale hydrogeological studies that have the objectives of constraining some key aquifer storage and hydraulic parameters [2, 10, 12] for better aquifer characterization.

In Ghana, erratic rainfall patterns have been noted in recent times. This has affected rain-fed agricultural activities in most areas. Efforts are being made at phasing groundwater resources at reasonable depths to meet growing domestic water needs whilst exploring the possibilities of developing the same resources for large-scale irrigation activities to augment agricultural activities. This would enhance food security and ultimately contribute to poverty reduction in the rural farming communities. Towards achieving these objectives, detailed hydrogeological investigations have been proposed to effectively characterize aquifers for proper development of the resource. This study forms one of the initial stages of this effort and utilizes a steady state numerical groundwater flow model to characterize the spatial variations in the hydraulic conductivities of a crystalline basement aquifer in southwestern Ghana. In addition to providing a conceptual framework of the groundwater flow system in the local environment, the methodology is of international importance as the utility of groundwater models in such endeavours can be applied in other terrains.

## 2. The Study Area

**Figure 1:**
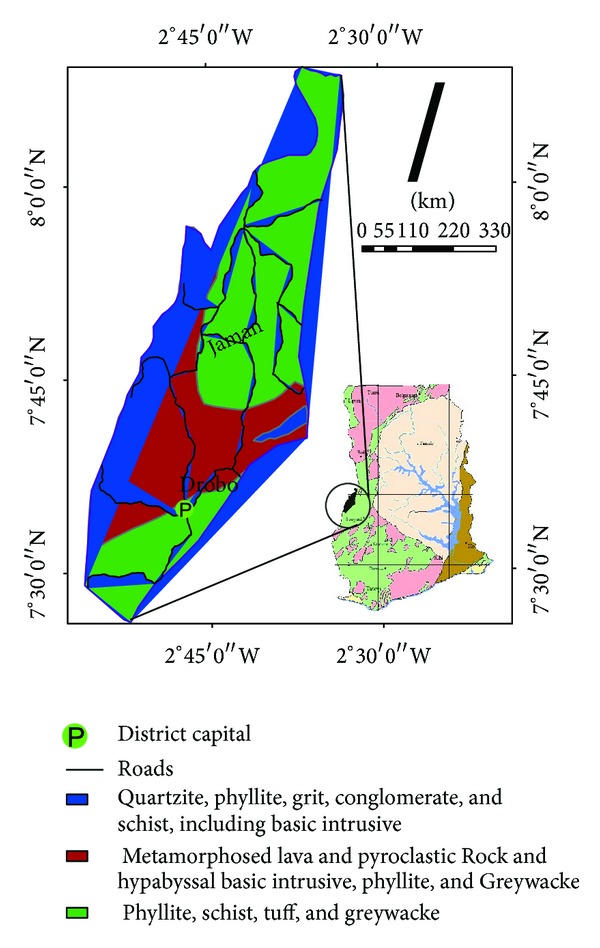
Map of the study area showing the geology.

The study area (Figure 1) is characterized by two main rainy seasons referred to as major and minor cropping seasons (April–July) and (September–November). It is characterized by a tropical climate, with high temperatures averaging 23.9°C (75°F) and a double maxima rainfall pattern [13]. Rainfall ranges from an average of 1000 mm in the northern parts to 1400 mm in the south. The entire domain is drained by tributaries of the Volta river basin [14]. The major economic activity there is agriculture which employs the majority (about 66%) of the economically active population. Cashew, coffee, teak, rubber, and tobacco are the main cash crops in the area [14].

The area is underlain by aquifers of the Birimian Province [15], which is one of the five proposed hydrogeological provinces in Ghana. It is characterized by basaltic and andesitic lavas, pyroclastic rocks, hypabyssal intrusive rock, and greywacke [16]. The basic volcanic and pyroclastic rocks have been largely altered to chloritised and epidotised rocks that are sometimes loosely referred to as greenstones. Generally, rocks of the Upper Birimian are intensely folded with steep dips and general north-south or north-west strikes [17]. In the absence of primary permeabilities, the rocks owe their hydrogeological conditions to the presence and pervasiveness of secondary structural entities which are diverse amongst rocks of the Birimian Province. Available hydrogeological data suggest that aquifers of the Birimian Province are amongst the most prolific in the country. Depths of boreholes drilled through rocks of the Province range between 35 m and 62 m with an average of 42 m [15]. Borehole depths in areas underlain by the granitoids are similar and range between 35 m and 55 m, with an average of 50 m [18]. In some areas, the regolith is tapped at relatively shallow depths with relatively shallow hand dug wells. Aquifer transmissivity of the productive zones of the Birimian Province ranges between 0.2 m^2^/d and 119 m^2^/d, with an average of 7.4 m^2^/d [15]. In these aquifers, storativity ranges between 0.003 and 0.008. Transmissivity within the regolith is slightly higher than that observed in the integrated aquifer system and ranges between 4 m^2^/d and 40 m^2^/d with an average of about 10 m^2^/d. For the integrated aquifer systems in the province, borehole yields are generally low and range from 0.48 m^3^/h to 36.4 m^3^/h with a mean yield of 7.6 m^3^/h. Differences in the degree of weathering within the granitoids probably account for the lower yields observed in these rocks.

## 3. Materials and Methods

### 3.1. Choice of Numerical Code

The flow of groundwater of constant viscosity and density through a heterogeneous, anisotropic porous medium under nonequilibrium conditions is described by (1) [19–21] which is obtained through the application of the Darcy law and the law of conservation of mass:

(1) $$\begin{array}{c} K_{x}\frac{\partial^{2}h}{\partial x^{2}}+K_{y}\frac{\partial^{2}h}{\partial y^{2}}+K_{z}\frac{\partial^{2}h}{\partial z^{2}}\pm W=S_{s}\frac{dh}{dt}, \end{array}$$

where *K*_*i*_, *W*, and *S*_*s*_, respectively, refer to the hydraulic conductivity in the *i*th direction, sources/sinks, and the aquifer specific storage.

This equation has been used in diverse forms to model groundwater flow depending on the prevailing conditions. For instance, where it cannot be safely assumed that density and viscosity are largely constant, (1) is modified to accommodate such a situation. Such a situation arises especially in coastal aquifers where saline water intrusion imposes variable salinities which in turn result in spatially variable groundwater densities. In such a case, a finite element code of the kinds of FEMWATER [22] which is based on the form of (1) which incorporates viscosity and density variations attending changes in the chemical constitution of groundwater in the space of a domain.

In the current study, steady state conditions were assumed. In this respect, the time variable nature of the hydraulic head on the right hand side of (1) was regarded negligible because the current sinks are not considered significant enough to cause such changes. In addition, a cursory analysis of the hydrochemical data from the terrain had suggested that groundwater density and viscosity would largely be the same throughout the domain. As such, (2) was used:

(2) $$\begin{array}{c} K_{x}\frac{\partial^{2}h}{\partial x^{2}}+K_{y}\frac{\partial^{2}h}{\partial y^{2}}+K_{z}\frac{\partial^{2}h}{\partial z^{2}}=0. \end{array}$$

On the basis of this, United States Geological Survey's Modular Finite Difference Groundwater Flow Modelling code, MODFLOW-2000 [23], was chosen to simulate the groundwater flow system in the study area. MODFLOW is arguably the most tested finite difference numerical code and has proven to accurately predict hydrogeological systems within the limits of field conceptualization and the available data. The Groundwater Modelling System, GMS [24], which contains MODFLOW and has an inbuilt graphical interface for MODFLOW output and a versatile GIS system for conceptualization purposes, was employed in this study.

### 3.2. Data Sources and Conceptualization

The hydrogeological data for this study were obtained from the offices of the Community Water and Sanitation Agency, CWSA, the SAL Consult (a hydrogeological consulting firm based in Accra), the Water Resources Commission, and the Geological Survey Department of Ghana. Data from borehole logs and the historical account of the geology and hydrogeology assisted in the vertical conceptualization of the terrain. The conceptualization was based on logs from 22 boreholes. The base map (the geological map of the terrain) was imported and registered in the GMS using the Map tools. Spatial discretization for the aquifer hydraulic parameters was facilitated by the local geology.

On the basis of the available data, the terrain was conceptualized as a single layer model. The lower limits of the aquifer system were conceptualized as a confining layer to coincide with the impervious material beneath. The upper limit was modelled as a convertible layer to coincide with semiconfining conditions prevailing in the area. In the absence of physical boundaries to limit flow on all sides, the vertical walls were all conceptualized as General Head Boundaries, GHB [25]. This would enable the simulation of net groundwater flow across the boundaries of the terrain. The GHB is a head dependent condition which simulates flow across a boundary based on the head difference across the boundary and the conductance of the material across the boundary (3). Consider

(3) $$\begin{array}{c} Q=C_{d}dh, \end{array}$$

where *Q*, *C*_*d*_, and *dh* are, respectively, the flow across the boundary, the conductance of the material, and the hydraulic head difference across the boundary.

An observation coverage was created to accommodate hydraulic data from 12 boreholes for the purpose of calibration. Coverages were similarly created for the hydraulic conductivity and recharge fields as well as the GHB condition. Data of the upper and lower limits of the aquifer were imported as text files as part of the conceptualization process. A grid system was then automatically developed to overlay the domain. A uniform rectangular grid system was used for the entire domain as the intended purpose was to general flow without focus on any particular location.

### 3.3. Numerical Simulation

The conceptual model was converted into a numerical MODFLOW model to begin the simulation. The thickness of the layer was defined by mapping the top and bottom elevations obtained from the borehole logs into MODFLOW. The appropriate solver and flow packages were then selected to begin the simulation of flow. An elaborate account of documentation of the MODFLOW code is contained in the US Geological Survey reports [23]. However, in the GMS system, the solver and flow packages are available in the Global options. Three flow packages are available: the Layer Property Flow (LPF), the Block Centered Flow (BCF), and the Hydrogeologic Unit Flow (HUF) packages. There are five solver packages: the strongly implicit procedure, the Pre-Conditioned Conjugate Gradient (PCG), the Slice Successive Over-relaxation (SOR), and the Geometric Multigrid (GMG) approach. In this study, the LPF and PCG were, respectively, chosen as the flow and solver packages to simulate the conditions in the terrain. The LPF package supports two types of layers: confined and convertible. In a confined layer, the transmissivity is constant throughout the simulation. The value of the transmissivity is computed from the hydraulic conductivity and cell elevations. Similarly, under transient conditions, the storage is computed by multiplying the specific storage by the cell elevation. A convertible layer is one in which the transmissivity varies with the hydraulic head throughout the simulation. The transmissivity is computed based on the cell thickness and the hydraulic conductivity and includes the possibility of a cell converting to a no flow boundary when the water level goes below the bottom elevation of the layer. In transient simulation (which was not simulated in this study) for a convertible layer, the storage contribution to flow is computed from confined and/or unconfined storage, depending on the level of the hydraulic head compared to the top elevation of cells [26]. The specific storage values are multiplied by cell volume to obtain confined storage capacity, whereas the specific yield is multiplied by the cell area to obtain unconfined storage capacity. Two storage capacities are therefore stored, and the program uses the appropriate value depending on head conditions. In this study, however, since the simulation was done under steady state conditions, this aspect of the package was not utilized as storage parameters are not assigned under steady state simulations. Since the aquifer system being simulated in this study is semiconfined, the convertible option was selected.

The Preconditioned Conjugate Gradient (PCG) solver [27] uses both outer and inner iterations. The user specifies the maximum number of iterations, so that the simulation terminates when the convergence criteria are not met within the maximum number of iterations. When the solver and flow packages were chosen, the model was set on forward run to begin the simulation.

### 3.4. Model Calibration

The only observation data used to calibrate the model were hydraulic head data as there are no known springs and other drains in the area. The calibration objective was therefore to ensure that the model computed hydraulic head data closely matched the observed data within a margin of error established during the conceptualization process. Manual calibration was first performed by altering the values of the hydraulic conductivity and recharge and running the model each time. When the model stabilized, the calibration was switched over to the automatic calibration mode through the Parameter Estimation, PEST [23, 24, 27, 28]. Parameter limits were defined for each of the coverages of recharge and hydraulic conductivity based on previous experiences in similar terrains and climate zones. The calibration target was set at ±2.5 m, which means that for each of the observation wells, calibration was said to have been achieved whenever the observed and model computed hydraulic heads were with 2.5 m of each other. In the PEST mode, the pilot point method [28, 29] was used for the hydraulic conductivity parameter, so that a smooth interpolation surface would be produced of the hydraulic conductivity field at calibration.

### 3.5. Sensitivity Analysis

Sensitivity analysis is recommended after calibration of every model. The objective is to test the stability of the model in the face of subtle variations in some of the key aquifer parameters. A highly sensitive model to any of the parameters is regarded as unstable and therefore not reliable for use in predicting scenarios. In this project, sensitivity analysis was performed automatically through the PEST. In this way, histograms would be generated at the end of the calibration to indicate parameter sensitivities.

### 3.6. Abstraction Scenarios

Since the model was conceptualized and calibrated under steady state conditions, it is not appropriate for modelling fluctuations in groundwater storage. However, it can be used in a limited fashion to evaluate recharge and abstraction scenarios. For each of the observation wells used in this study, the yields estimated during pumping tests were applied as the initial abstraction rates. In the scenario analyses, abstraction rates were increased by 10%, 20%, 50%, 100%, and 150% whilst maintaining recharge at the calibrated rates. In the next scenario, the recharge was deliberately reduced by 10%, 20%, and 30% whilst abstraction rates were increased at 10%, 20%, 50%, 100%, and 150%. This scenario was expected to simulate possible reduction in groundwater recharge rates due to decreasing rainfall amounts at the recharge areas.

## 4. Results and Discussions

### 4.1. The Groundwater Flow System

**Figure 2:**
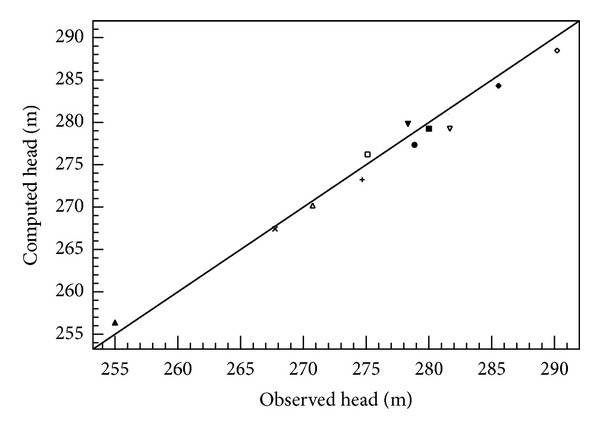
A linear plot showing the relationship between observed and computed hydraulic head.

**Figure 3:**
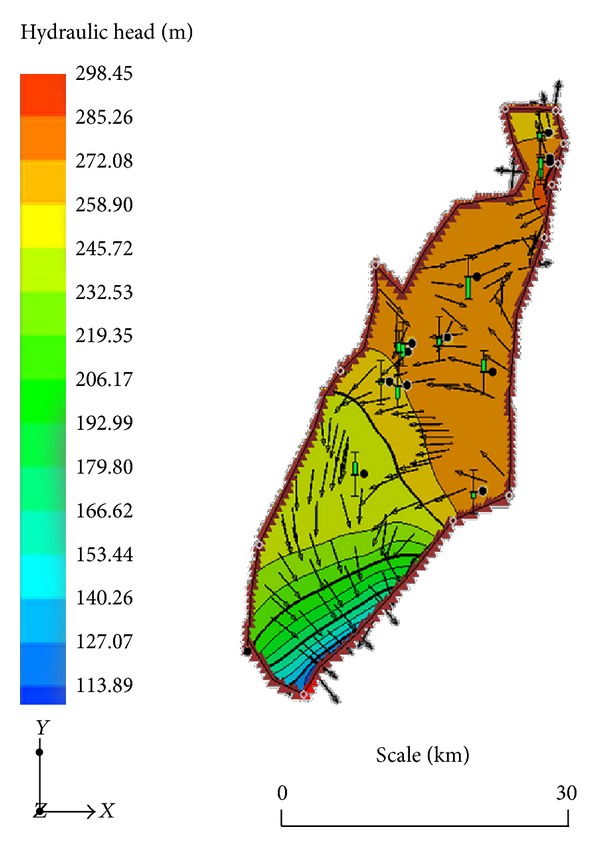
The potentiometric surface predicted by the model for the study area.

The model was deemed calibrated when the computed heads for all the wells were well within 2.5 m of the observed heads.  Figure 2 illustrates the relationship between the observed and model computed hydraulic heads for the 12 wells used for the calibration. A good match between model computed and observed hydraulic heads is obvious in Figure 2, suggesting that the model is reasonably calibrated within the limits of the data used and is therefore representative of the hydrogeological conditions prevailing in the terrain. The sensitivity analysis suggests that the model is largely stable to subtle variations in the key aquifer parameters of hydraulic conductivity and recharge. Since model calibration was achieved by varying the parameters of hydraulic conductivity and recharge, the ranges of values of these parameters at calibration were adjudged representatives of the conditions prevailing in the terrain. The predicted hydraulic head field is as presented in Figure 3 which suggests that the areas of the highest hydraulic head are in the northern parts of the terrain. However, there is no clearly defined direction of flow, indicating that the local structural entities that control the flow system are not oriented in any preferred direction. Local flow systems apparently dominate the flow in the area. Where the aquifer is highly heterogeneous with respect to the key aquifer hydraulic parameters, flow is largely haphazard and local flow systems are predominant. The same is true when the terrain is of considerably variable topography [20]. Heterogeneities in the hydraulic conductivity are more likely responsible for the observed pattern of flow in the current study.

**Figure 4:**
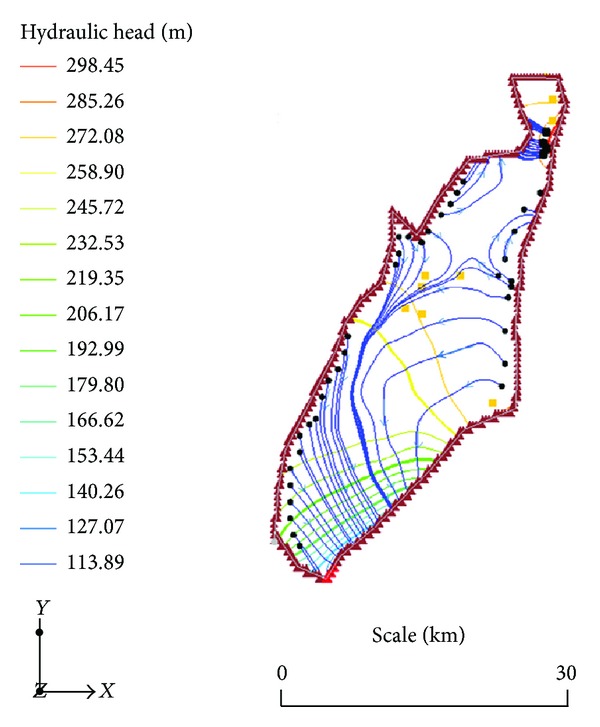
The most prominent groundwater flowpaths in the study area.

Five prominent flow paths have been defined in the study area (Figure 4), through detailed particle tracking from MODPATH [30]. There is a conspicuous flow divide in the north-central parts of the area. This is a hydraulic boundary to flow attributed mainly to aquifer heterogeneity rather than topographical complexities in the terrain. Although local topography has an influence on flow systems [1, 19] and can sometimes be used to predict the direction of flow, the ultimate determinant of the flow is the level of heterogeneity of the material of the aquifer. Where the topography is rugged and the aquifer is largely homogeneous and isotropic, the system is deficient in local flow systems and regional flow systems are dominant. On the other hand, where the surface topography is largely flat, marked heterogeneities of the aquifer can impose several complex local systems due to high levels of refraction of groundwater flowpaths through materials of contrasting hydraulic properties. The significance of this lies in the fact that where the surface topography alone is used to predict the trajectory of a particle in groundwater for the purpose of assisting in remediation purposes, the interventions can be misdirected if detailed analyses are not performed to culminate in the simulation of the flow. In all cases of groundwater resources development and solute transport studies therefore, numerical flow modelling is inevitable. The detail of the flow system is a relevant prerequisite for the effectual management of the resource.

Yidana et al. [9] predicted a general NE-SW groundwater flow pattern amongst similar crystalline basement aquifers in the south of the terrain using a similar methodology. The obvious departure in the current study suggests that secondary permeabilities in the Birimian Province are inherently diverse and do not necessarily align in a particular direction. Thus, whereas the regional structural geology suggests the dominance of NE-SW trending structural entities [16], local groundwater flow conditions may considerably deviate from this regional pattern, especially where extensive weathering occurs to enhance the local hydrogeology. Previous observations in other parts of Ghana [9–11] suggest that where the flow system is dominated by regional systems, a prominent NNE-SSW flow system is observed. The regional hydrogeology of Ghana therefore appears to be largely controlled by the regional structural grain. This finding implies that where the hydrogeology of an area is based on secondary permeabilities, variable groundwater flow patterns, resulting from the spatial variabilities of the entities controlling, the hydrogeology should be expected. A similar model within a granular unconfined aquifer system in the Keta Basin [26] produced a much more smooth flow pattern due to the dominance of primary permeabilities and the interconnectivities of such entities. The use of numerical techniques in this way is important towards enabling a proper understanding of mechanisms of groundwater flow and will facilitate the management of aquifers and groundwater resources.

### 4.2. The Hydraulic Conductivity Field

**Figure 5:**
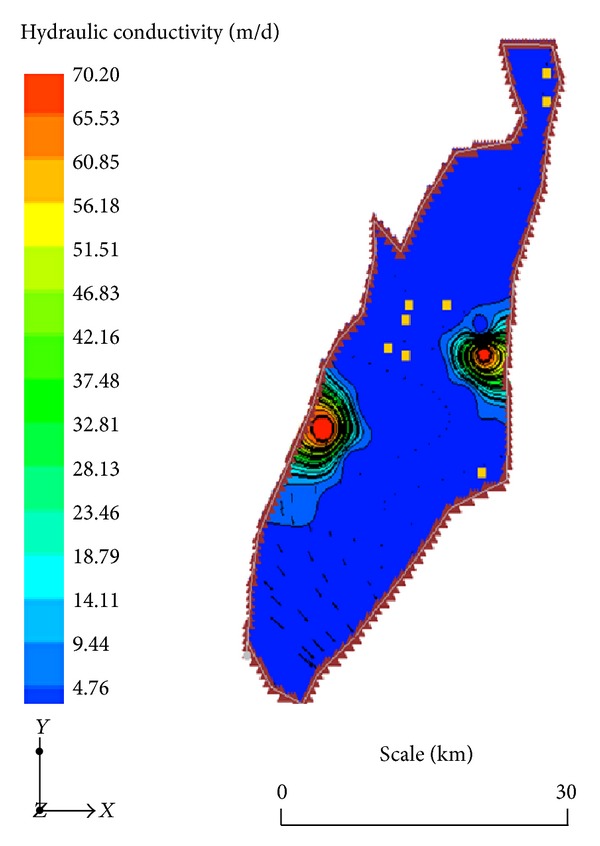
Calibrated hydraulic conductivity field for the study area.

A smooth map of the horizontal hydraulic conductivity field in the domain has been established through the pilot point method [27–29] in this study. It suggests a significantly heterogeneous system where hydraulic conductivity ranges between 4.5 m/d and over 70 m/d (Figure 5) and amply explains the observed groundwater flow pattern. There is no obvious relationship between the hydraulic conductivity field and the general distribution in hydraulic head (Figure 3). This suggests that (i) local variations in the vertical hydraulic conductivities which control vertical groundwater recharge in the area are significantly different from the patterns of the horizontal hydraulic conductivity and/or (ii) subsurface flows are the major sources of groundwater recharge in the terrain. The computed horizontal hydraulic conductivities in this study are largely consistent with the aquifer transmissivity data obtained from pump tests within the Birimian Province and reported in other publications [15] and is largely in sync with the general character of fracture controlled aquifer systems. The validity of hydrogeological data on aquifer parameters from pump tests in Ghana has been the subject of debate on grounds of speculations of partial penetration of boreholes and the concomitant effects on the results of pump tests. Generally, the use of pump-test estimates of aquifer parameters in regional hydrogeological investigations has been considerably criticized in the literature [31–33]. Razack and Huntley [31] and Huntley et al. [32], for instance, proved that such analytical techniques have the propensity to underestimate aquifer transmissivity values in heterogeneous alluvial aquifers and overestimate the same in fracture controlled aquifer systems. Mace [33] proposes empirical relationships between aquifer transmissivity and specific capacity for the estimation of the former, given data of the latter. Such empirical relationships have been established for some aquifers in Ghana [34, 35]. Other researchers have proposed the use of cokriging and the strength of the relationship between aquifer transmissivity and specific capacity to produce smooth prediction maps for the former. However, since such maps would be the product of data from pump tests estimates, they suffer the same defects in terms of reliability. Generally, aquifer transmissivity estimates from pump tests provide the character of the aquifer at the well scale alone and must therefore be used cautiously at the regional scale. This is because measures of aquifer permeability are scale dependent, and therefore point estimates may be misleading. In order to sufficiently characterize regional aquifers through the conventional pump tests alone, a very large number of such tests will have to be conducted. This is highly impracticable due to the prohibitive costs of such endeavours. It is on the basis of this fact that regional numerical groundwater flow models of the kind used in the current study have been proposed for such estimates. Numerical models by their nature predict aquifer parameters based on set of observed data and boundary conditions. They therefore largely predict the regional balance of these parameters fairly and accurately.

### 4.3. Groundwater Recharge and Resource Sustainability

Estimated vertical groundwater recharge in the area ranges between 3 mm and 109.5 mm per annum, equivalent to 0.25% and 9.13% of the average annual precipitation in the basin. Apparently, a significant proportion of the total groundwater input in the terrain results from subsurface flows through the GHB condition, contributing to over 35,000 m^3^/day of the total groundwater input into the system. Contributions of subsurface flows are largely responsible for the observed groundwater flow geometry in the study area. This much of recharge holds significant promise for groundwater resources development in the area. The estimated rates are compatible with the estimates of Yidana et al. [9] in the southern parts of the terrain using the Chloride Mass Balance, CMB, technique and model calibration. It is also in agreement with the general trend observed in other parts of the country where recharge had been estimated through a variety of other methods [11, 18, 26]. However, since transient conditions were not simulated, changes in groundwater storage could not be estimated. The application of groundwater flow modelling in hydrogeological studies and groundwater resources assessment has not been popular in Ghana and much of the West African subregion, where inaccessibility to potable water resources sometimes constitutes one of the major causes of extreme deprivation.

### 4.4. Scenario Analyses

Freeze and Cherry [1] defined the concepts of sustainable aquifer and basin yields to reflect the maximum permissible abstraction rates from all wells through the aquifer and basin, respectively. The need for clear definitions of these concepts stem from the fact that most groundwater resources studies aim at determining the maximum abstraction rates that are compatible with the hydrogeological environment. The definition of the exact abstraction rates that are regarded as optimal is dependent on several considerations such as the number of intervening processes and/or factors that have a bearing on groundwater resources. In this study, sustainable aquifer yield is defined as the maximum possible groundwater abstraction rates that will lead to minimal effects on the general distribution of the hydraulic head. At the current calibrated groundwater recharge conditions, the model suggests that the aquifer can sustain increment in groundwater abstraction rates up to 200% of the current rates with minimal (less than 15%) depreciation in the hydraulic head throughout the entire domain. This is in keeping with the observed recharge rates and the apparently relatively pristine groundwater abstraction conditions prevailing in the area. The implication is that whereas the current recharge rates may not be guaranteed due to possible climate change impacts, the aquifer holds significant fortunes for large-scale abstraction to support irrigation schemes. The second scenario assessed the impacts of increased groundwater abstraction rates whilst recharge is reduced from the current rates. This study assessed reduction in recharge by 10%, 20%, and 30% of the current levels. The results of the simulation suggest that there will be an appreciable depreciation in the hydraulic head leading to possible reversals in the flow pattern when recharge is reduced by up to 30% whilst abstraction rates continue to increase by up to 150% of the current levels. The assumption of steady state conditions in the current study was based on the fact that the current abstraction rates are considered very minimal and insignificant. This has been amply justified through the simulation of the various scenarios of groundwater abstraction rates in this study.

## 5. Conclusions

This research proves that model calibration is one of the best approaches towards constraining the spatial distribution of aquifer hydraulic parameters in large-scale regional hydrogeological assessments. Therefore, in regional hydrogeological studies, the use of numerical groundwater models is very much recommended for achieving the goals of aquifer characterization. In this study, a hydraulic conductivity field has been developed for a crystalline basement aquifer in Southern Ghana through model calibration. It suggests that aquifer hydraulic conductivity in the area ranges between 4.50 m/d and over 70 m/d. The heterogeneity in the predicted dataset appears to be dictated by the heterogeneities in the structural entities that govern the hydrogeological properties of the aquifers in the area. Local flow systems appear to be predominant due to the observed heterogeneities in the aquifer properties in the area. Five prominent flow lines have been identified in the study area where groundwater recharge rates range between 0.25% and 9.13% of the total annual precipitation in the area. A substantial proportion of this recharge appears to accrue from subsurface flows. This much recharge holds promise for large-scale development of groundwater resources for irrigation in the area as suggested by the results of the various scenarios of groundwater abstraction simulated in the terrain. Even with a reduction in recharge by up to 30% of the current rates, the domain can sustain an increase in groundwater abstraction by up to 150% of the current abstraction rates. Evaluation of the effects of climate change/variability on groundwater resources sustainability and livelihood will require the use of groundwater flow models especially when such models are calibrated for transient conditions.
